# Very Few RNA and DNA Sequence Differences in the Human Transcriptome

**DOI:** 10.1371/journal.pone.0025842

**Published:** 2011-10-12

**Authors:** Daniel R. Schrider, Jean-Francois Gout, Matthew W. Hahn

**Affiliations:** 1 Department of Biology, Indiana University, Bloomington, Indiana, United States of America; 2 School of Informatics and Computing, Indiana University, Bloomington, Indiana, United States of America; University of Montreal, Canada

## Abstract

RNA editing is an important cellular process by which the nucleotides in a mature RNA transcript are altered to cause them to differ from the corresponding DNA sequence. While this process yields essential transcripts in humans and other organisms, it is believed to occur at a relatively small number of loci. The rarity of RNA editing has been challenged by a recent comparison of human RNA and DNA sequence data from 27 individuals, which revealed that over 10,000 human exonic sites appear to exhibit RNA-DNA differences (RDDs). Many of these differences could not have been caused by either of the two previously known human RNA editing mechanisms—ADAR-mediated A→G substitutions or APOBEC1-mediated C→U switches—suggesting that a previously unknown mechanism of RNA editing may be active in humans. Here, we reanalyze these data and demonstrate that genomic sequences exist in these same individuals or in the human genome that match the majority of RDDs. Our results suggest that the majority of these RDD events were observed due to accurate transcription of sequences paralogous to the apparently edited gene but differing at the edited site. In light of our results it seems prudent to conclude that if indeed an unknown mechanism is causing RDD events in humans, such events occur at a much lower frequency than originally proposed.

## Introduction

The accurate transcription of genomic DNA into RNA is essential for carrying out cellular processes, as RNA transcripts are either translated into functional proteins or perform functions directly. However, in humans [1], plant chloroplasts and mitochondria [2], and certain viruses (e.g., [3]), there are known cases of RNA transcripts differing from the transcribed DNA at specific positions. For example, in humans, adenosine deaminases acting on RNA (ADARs) replace certain adenosines (A) with inosines, which then act as guanosines (G) during translation [4], [5]; furthermore, the protein APOBEC1 causes a small number of C→U changes [6], [7], [8]. Many of these RNA editing events result in alternative proteins that are useful to the organism, and alterations of the frequency of certain RNA editing events can negatively affect organismal function [9].

Despite the demonstrated benefits of RNA editing events, RNA editing is currently viewed as a relatively rare phenomenon, with one comprehensive study identifying only several hundred A→G changes in the human transcriptome [10]. However, a recent study comparing RNA and DNA sequences from 27 human individuals challenges this view [11]. In this study, Li et al. [11] discovered more than 10,000 human exonic sites where the RNA sequences appeared to differ from DNA sequences obtained from the same individual. Interestingly, the majority of these RNA-DNA differences (RDDs) produce changes other than the typical A→G or C→U changes expected by known mechanisms of RNA editing [6], [7]. This surprising result implies that most RDDs are produced by some as yet unknown molecular mechanism. Perhaps even more strikingly, this study found a much larger number of modified sites in human mRNAs (10,210) than any study to date, suggesting that RDDs are an important contributor to transcriptomic diversity.

Li et al. [11] experimentally confirmed that many of these modified RNA sequences do exist and sometimes result in altered proteins, and are therefore not artifacts of sequencing error. Furthermore, by restricting their analysis to mostly invariant sites, they minimized the likelihood that unsampled genetic variation at the RDD site could result in false positives; comparison with previous studies also ensured the accuracy of their genotype calls at each RDD site. However, the authors did not take adequate steps to ensure that the modified RNA could have resulted from accurate transcription of DNA somewhere else in the genome. Their only check of the DNA sequences present in each individual was to ensure that RNA-seq reads mapped uniquely to the annotated human GENCODE mRNA sequences [11]. Unfortunately, this step is not enough to ensure the absence of genomic sequences matching the modified sequences. For example, spurious RDDs would be observed if a highly similar paralog absent from GENCODE and differing from the edited locus at the RDD site was transcribed and translated. In this case the RNA-seq reads supporting RDD events could be derived from sequences other than the seemingly modified gene.

Because Li et al. only searched their RNA-seq reads against the GENCODE sequences, there are actually three different potential sources of spurious RDDs. First, transcribed sequences paralogous to a GENCODE gene present in the reference genome but not included in the GENCODE predictions of protein-coding genes would be incorrectly inferred to be an RDD if nucleotides differed between the two sequences. Second, even ensuring that sequences are unique in the reference genome does not ensure that they are the result of RNA editing: paralogous sequences present in the reference genome that contain a single-nucleotide polymorphism in the sampled individual (such that the exact sequence is not present in the reference genome) would also appear to be RDDs. Finally, nucleotide differences in segregating copy number variants [12] that are absent from the reference assembly and contain a single nucleotide difference from their paralogous sequence in the reference genome would also be inferred to be RDDs. In any of these cases the paralogous sequence, if transcribed, has the potential to confound the analysis by producing evidence of post-transcriptional modifications where none exist. We examined both the DNA and RNA sequences analyzed by Li et al [11], and found that the vast majority of apparent RDD sites identified in their study match genomic sequence and are therefore most likely the result of accurate transcription of paralogous sequence rather than some unknown RNA editing mechanism.

## Results

In order to determine the extent to which RDD events could be erroneously called due to transcription of paralogous sequences matching RDD sites, we first asked whether RDD calls made by Li et al. [11] matched sequence elsewhere in the genome by searching their 10,210 RDD sites against the reference genome, a step not taken in the original paper. When extracting RDD sites and flanking sequences from the reference genome in order to perform this search, we noticed that at 39 of these RDD sites the reference genome exhibited the nucleotide reported by Li et al. to be present in the mRNA but not in the genome (which we will refer to as the “RDD nucleotide”). This suggests that these 39 RDD events were reported in error. We then searched the remaining 10,179 RDD sites against the reference genome (see Materials and Methods) and found that 890 of these RDD sites have a paralog in the reference genome that exhibits the RDD nucleotide. The observation of RNA-DNA sequence differences at these sites suggests that the inferred RDDs are more likely due to transcription of these paralogous sequences than RNA alterations. This explanation is supported by the fact that 674 (75.7%) of these paralogs are found in transcribed regions of the genome, and 640 (71.9%) are located within an annotated gene (Materials and Methods).

We also found that 1,316 additional RDD sites have at least one paralog in the reference genome not containing the RDD nucleotide. However, such paralogs could contain polymorphisms such that the transcription of these sequences would result in the appearance of RDDs, if the polymorphic allele not present in the reference genome matches the RDD nucleotide. Again, this possibility is supported by the large percentage of such paralogs found in genes (86.5%) or transcribed regions of the genome (86.2%). In total, RDD sites are much more likely to have a paralog than an average human gene (80.6% of RDD sites versus 68.3% of all human genes; *P*<2.2×10^−16^; Fisher's Exact Test using paralogy assignments from ref. [13]). In addition to paralogs present in the reference genome, duplication polymorphisms absent from the reference genome could also create the appearance of RNA-DNA differences. This possibility seems especially relevant given that 3,893 of the remaining RDD sites are either within a duplication listed in the Database of Genomic Variants [14] or have a paralog within such a duplication—a 1.5-fold enrichment of RDD sites for copy number-variable regions of the genome (*P*<0.001; see Materials and Methods).

For both of the above possibilities to explain the appearance of RDDs, there must be genomic DNA present in an individual (and not the reference genome) that matches the RDD nucleotide. We therefore asked whether Li et al.s' RDD calls for each individual were matched by genomic reads from the same individual, again, a step not taken in the original paper. Because the list of individuals exhibiting each RDD site was not made available, we attempted to recapitulate Li et al.'s results by mapping their RNA-seq data to a database of transcripts containing the 10,210 RDD sites. We used the short-read mapping program BWA [15] to map all RNA-seq reads and applied Li et al.'s criteria for detecting RDD events and determining which events occur in which individuals (Materials and Methods). For most individuals, the number of RDD events we called closely matched the corresponding number of events found by Li et al. (compare our Supplementary Figure S1 with Figure 1B from Li et al.—the exact number of events originally found in each individual was not provided by the authors), suggesting that we fairly accurately recreated their set of RDDs. Next, we mapped genomic reads from these same individuals available through the 1000 Genomes project [16] to these RDD sites, and found that on average 30.5% of RDD events called in an individual are matched by at least one genomic read from that same individual containing the RDD nucleotide. This result suggests that a substantial proportion of RDD sites called by Li et al. may not be the result of some type of RNA editing event. Instead, there are likely paralogous sequences matching the RDD nucleotide in some or all of the 27 individuals, and these apparently edited transcripts could be the result of transcription of these sequences.

**Figure 1:**
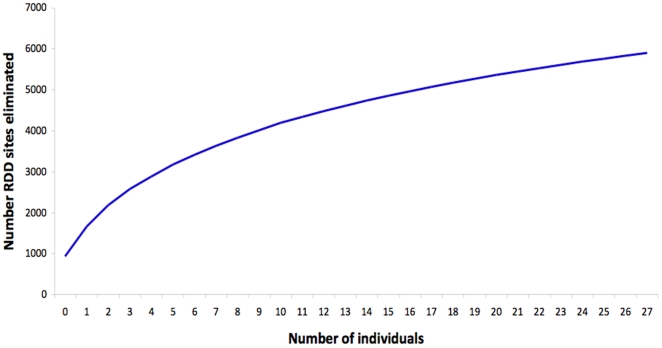
The number of RDD sites eliminated increases with number of individuals examined. This graph was generated by randomly adding an individual's genomic sequence data to the total dataset and counting the number of RDD sites eliminated (i.e. found to match genomic sequence) once that individual was added. This was repeated 1,000 times and the average number of eliminated RDDs was calculated for each number of individuals. The value for zero individuals is 929 because this is the total number of RDDs that can be eliminated solely by examining the reference genome.

Given the low genomic sequencing coverage of many of the 27 individuals [16], we suspected that even more of Li et al.'s RDD sites could have been false positives. We reasoned that if an RDD site matched genomic sequence from any individual, whether that individual met the criteria for exhibiting the specific RDD event or not, the RDD site was likely not a true editing event. We therefore examined genomic reads from all individuals to determine how many RDD sites matched genomic sequences present in this sample. In total, we found that 74% of RDD sites have at least one genomic read matching the RDD nucleotide in at least one individual. Because some of these matches could be due to simple sequencing errors in genomic reads, we used more stringent criteria to identify a higher-confidence set of genomic sequences, and examined the numbers of reads not matching either the genomic nucleotide or the RDD nucleotide to verify that sequencing error had a minimal impact on this analysis (Materials and Methods). These methods found that the majority (5,666 or 55%) of the 10,210 RDD sites match genomic sequence from at least one individual. In total, 5,900 (57.8%) of the 10,210 RDD sites match either sequence from one of the 27 individuals or from the human reference genome. Table S1 provides a list of the 10,210 RDDs, and whether or not we find evidence for a genomic explanation for the event.

If RNA editing is largely restricted to A→G substitutions, and if the 5,900 RDD sites matching genomic sequence data are truly spurious, then the remaining 4,310 RDD sites in Li et al.'s set should be enriched for A→G changes. This is indeed the case, as the percentage of all RDD sites that are A→G differences increases from 22.8% to 23.5% (*P* = 0.013; Fisher's Exact Test) when RDDs matching genomic sequence are removed from the set. When all RDDs matching at least one genomic read from any individual are removed, the percentage of A→G events grows to 24.5%, though this increase is not statistically significant. While this strongly suggests that the RDDs matching genomic sequence are spurious, a substantial number of non-A→G events remain. This observation can be interpreted in two ways: either the remaining RDD sites are likely true RNA editing events, or they are also erroneous observations due to accurate transcription of genomic sequences missed because of the relatively low coverage of the sequences examined in our analysis. The latter interpretation is supported by Figure 1, which shows the growth in number of RDD sites matching genomic sequence according to our criteria when examining sequence data from additional individuals (Materials and Methods). Extrapolating from this curve reveals that over 90% of all 10,210 RDDs would be removed by our analysis using sequence data of comparable quality and coverage from 245 individuals. It therefore seems prudent to conclude that many of the 4,310 remaining RDDs could have been observed in error due to transcription of paralogous loci, and that the vast majority of the 10,210 RDD sites called by Li et al. can be explained by faithful transcription of genomic DNA.

## Discussion

Our results suggest that by failing to consider the totality of genomic data from their sample population and paralogous loci present in the human reference genome, Li et al. [11] grossly overestimated the amount of RNA editing in the human genome. However, just as striking as the number of RNA-DNA differences described in their paper was the distribution of these changes—the vast majority of RDD sites they report are not A→G or C→T substitutions, and therefore could not have been caused by any known editing mechanism. Notably, a recent study examined DNA and RNA sequences from a different sample of 15 individuals and did not find either of these striking results [17]. Using a similar method but with more stringent criteria for detecting RNA-DNA differences, this study reports a much smaller number of RDD sites (1,809) with over 50% of these due to A→G events—a distribution far more in line with expectations based on current knowledge of RNA editing than the results reported by Li and colleagues. Indeed, the removal of apparently spurious RDD sites from Li et al.'s set of RDDs alters the distribution of editing events in this direction.

Although we are not able to explain all 10,210 RDD sites using genomic sequence, this is in large part due to inadequate sequence coverage, as shown in Figure 1. Furthermore, a substantial fraction of RDDs that Li et al. attempted to validate experimentally were not confirmed. For example, of the experiments conducted to validate RDD events, the one that attempted to confirm by far the largest number, searching 120 RDD sites against the more than 8 million human ESTs in GenBank, confirmed only 81 (67.5%) of these events [11]. While the failure to confirm some of the RDD events is probably due to the difficulty in sequencing mRNAs expressed at low levels, it is also likely that some unconfirmed RDDs were due to sequencing error in the original RNA-seq data. It is therefore unnecessary to invalidate each of the 10,210 RDD sites reported by Li et al. using data from genomic DNA in order to cast doubt on their conclusion that there is a novel and widespread mechanism of RNA editing, at least until stronger evidence is provided. Taken together, the results from Li et al. [11], Ju et al. [17], and ourselves are not inconsistent with some unknown mechanism of RNA editing acting on the human transcriptome at a much smaller number of sites. However, more work will be required to precisely determine the frequency of any such editing events, if they are indeed occurring, and the mechanism(s) responsible for these events.

## Materials and Methods

### Searching RDD sites against the reference genome

We extracted each of Li et al.'s 10,210 RDD sites [11] as well as 49 bp of flanking sequence on either side of the edited site from the NCBI 36 human reference genome. 39 of these were found to exhibit the same nucleotide in the reference genome as in the edited state as reported by Li et al., and were excluded from further analysis. We then used BLAT [18] to search the remaining 10,171 against the reference genome using default *repMatch = 100000* and *stepSize = 5*. Only BLAT hits of greater than 90% identity were considered. A hit was considered to be a paralog capable of producing a spurious signal of RNA-DNA sequence difference if it was at least 50 bp long (the length of the RNA-seq reads used by Li et al. [11]), and matched the nucleotide present in edited transcripts but not the genome at the RDD site. 890 RDDs were found to have such a paralog. Hits at least 50 bp long but not matching the edited nucleotide were considered paralogs not exhibiting the RDD state in the reference genome. 1,316 RDDs were found to have such a paralog.

To determine whether a site paralogous to an RDD site was found within an annotated gene, we downloaded the coordinates of all annotated human genes from version 54 of Ensembl [19], and determined which sites paralogous to RDDs were found within these coordinates. Similarly, we downloaded mapping results of 296,734 mRNA sequences to the human genome from the UCSC genome browser [20], and determined which RDD paralogs were found within regions matching these sequences.

### Finding RDD sites or paralogous sequences in duplication polymorphisms

To determine which RDD sites or paralogs of RDD sites were found within regions known to have duplication polymorphisms, we downloaded version 10 of the Database of Genomic Variants [14]. We then examined all copy number variants exhibiting gain alleles and found that 3893 of RDD sites or their paralogs were found in such regions. To determine whether RDDs and their paralogs were significantly overrepresented in copy number variants, we replaced each of the RDD sites or paralogous sites examined with a random position in the human genome, and checked whether this position was found within a duplication polymorphism. We repeated this process 1,000 times, and never found as many random positions in duplications as RDD or paralogous positions. Thus, RDD sites are significantly biased toward copy-number variable regions of the human genome exhibiting duplication alleles.

### Using RNA-seq to call RDD sites

In order to determine which individuals exhibit each of the 10,210 RDD sites reported by Li et al. [11], we downloaded their RNA-seq data and mapped these reads to a database of transcripts containing the 10,210 sites using BWA [15]. In the original paper, Bowtie [21] was used to map RNA reads to the GENCODE genes. However, BWA is more accurate and allows for indels [15], so we use it here. In any case, if RDDs are common, then their detection should be robust to changes in alignment software. Following Li et al. [11], we only examined reads mapping to a given RDD site with no more than two mismatches. We then used their criteria to determine which individuals exhibit each RDD site. Briefly, in order for a position in an individual to be categorized as an RDD event in which the genomic nucleotide A is replaced with nucleotide B in the messenger RNA, Li et al. require 1) that at least 10 RNA-seq reads map to the site, 2) that at least 90% of these reads match either A or B, 3) and that of the reads matching either A or B, at least 10% match nucleotide B.

### Finding genomic reads matching RDD sites

Genomic reads from the same 27 individuals examined by Li et al. [11] were downloaded from the 1000 Genomes Project [16] website. These reads correspond to a mix of 454, Illumina, and SOLiD technologies. We constructed a database of all RDDs by extracting from the reference human genome (hg18) 49 nucleotides on each side of all RDD sites, using the position and strand information in Table S10 from Li et al. This database was indexed for search with BWA [15], using the ‘is’ parameter for the algorithm (option –a is). A color-space version of this database was built for mapping of SOLiD reads. We used BWA to map the genomic reads to this database, initially allowing up to 5 mismatches (option –n 5) and no gaps (option –o 0). Reads having more than one mismatch other than those corresponding to the edited nucleotide at RDD sites were eliminated from the remainder of the analysis.

### Controlling for sequencing error

We initially found that 74% of RDD sites had at least one genomic read in at least one individual matching the edited nucleotide. However, nearly 54.6% of RDD sites had a genomic read matching neither the genomic nucleotide nor the edited nucleotide, which may be due to sequencing error. Therefore, to minimize the effect of sequencing error, we asked how many RDD sites had at least 90% of genomic reads from an individual matching either nucleotide A (the genomic nucleotide) or nucleotide B (the edited nucleotide) and at least 10% of these reads matching nucleotide B. Note that these requirements are similar to those used by Li et al. and ourselves to call RDD sites. (The only relevant requirement omitted for this step is that the total coverage at a site be greater than or equal to 10, which would greatly reduce our sensitivity given the lower coverage of the genomic sequence data.) We found 5,666 RDD sites (55.5%) meeting these criteria. To ensure that this approach had adequate specificity, we also checked how often these criteria were met by reads matching one of the other two nucleotides. In other words, when we randomly replaced the B nucleotide of an RDD site with one of the two nucleotides not equal to A or B and repeated our test, we found only ∼910 RDD sites (8.9%) meeting these criteria (mean after 10,000 iterations). Similarly, we verified that sequencing error was not responsible for the sizeable proportion of RDD sites present in a given individual that were also matched by at least one genomic read from that same individual. While on average 30.5% of RDD events found in a given individual were matched by a genomic read from that same individual containing the B nucleotide, only 8.2% of RDD events matched a genomic read having one of the other two nucleotides. It should be noted that some reads not matching either the A or the B nucleotide may be the result of additional polymorphisms or paralogs rather than sequencing error. Thus, we may be overestimating the impact of sequencing error.

## Supporting Information

Figure S1 **The number of RDD events found in each individual.** We called RDD sites we found using RNA-seq data from each of the 27 individuals after performing our own mapping and reapplying the original criteria used by Li et al. [11]. Since the number of RDD events originally called in each individual was not made available, the only way to compare the similarity of our RDD calls in each individual with the original calls is by comparing this figure to Figure 1B from Li et al [11].
(TIF)

Table S1 **Detailed list of the 10,210 RDD sites.**
(XLS)
