# Supplementary figures and images for: Very Few RNA and DNA Sequence Differences in the Human Transcriptome
Source: PLoS One. 2011 Oct 12;6(10):e25842. doi: 10.1371/journal.pone.0025842 (PMC3192132; doi:10.1371/journal.pone.0025842)

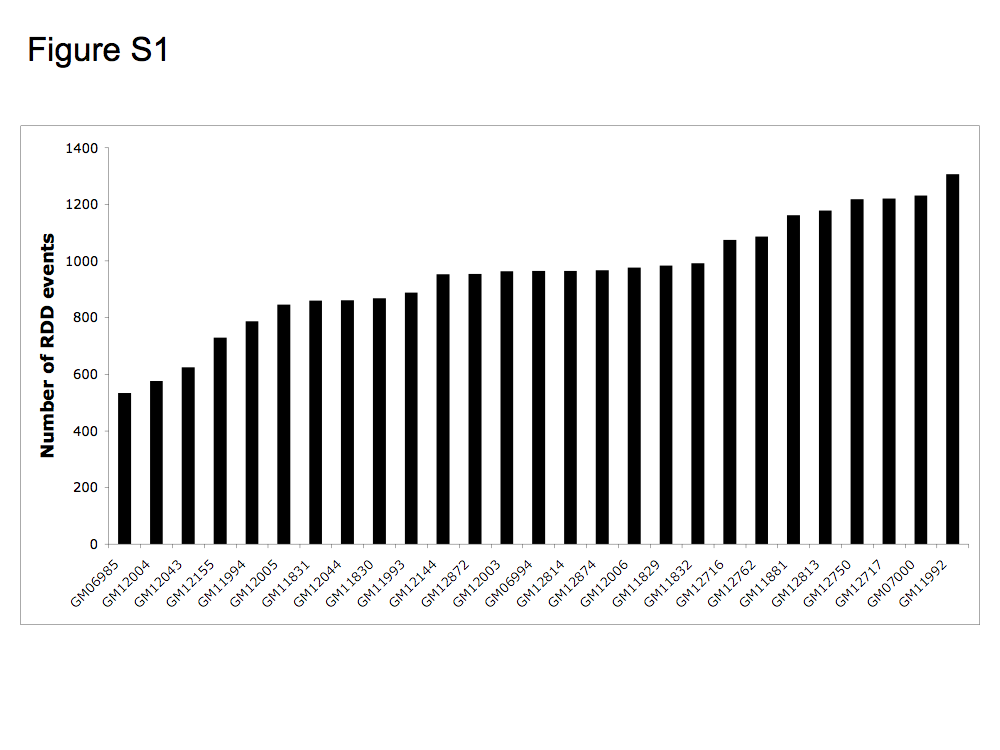

Supplement: Figure S1 — The number of RDD events found in each individual. We called RDD sites we found using RNA-seq data from each of the 27 individuals after performing our own mapping and reapplying the original criteria used by Li et al. [11]. Since the number of RDD events originally called in each individual was not made available, the only way to compare the similarity of our RDD calls in each individual with the original calls is by comparing this figure to Figure 1B from Li et al [11]. (TIF) [file pone.0025842.s001.tif]
